# Testosterone deficiency impairs cardiac interfibrillar mitochondrial function and myocardial contractility while inducing oxidative stress

**DOI:** 10.3389/fendo.2023.1206387

**Published:** 2023-09-13

**Authors:** Patrícia Ribeiro do Val Lima, Karoline Sousa Ronconi, Elis Aguiar Morra, Paula Lopes Rodrigues, Renata Andrade Ávila, Eduardo Merlo, Jones B. Graceli, Maylla Ronacher Simões, Ivanita Stefanon, Rogério Faustino Ribeiro Júnior

**Affiliations:** ^1^ Department of Physiological Sciences, Federal University of Espirito Santo, Vitoria, ES, Brazil; ^2^ Department of Morphology, Federal University of Espírito Santo, Vitoria, ES, Brazil

**Keywords:** testosterone, mitochondria, cardiac contractility, subsarcolemmal, interfibrillar, mitochondrial subpopulations, oxidative stress

## Abstract

**Introduction:**

Clinical studies have shown that low levels of endogenous testosterone are associated with cardiovascular diseases. Considering the intimate connection between oxidative metabolism and myocardial contractility, we determined the effects of testosterone deficiency on the two spatially distinct subpopulations of cardiac mitochondria, subsarcolemmal (SSM) and interfibrillar (IFM).

**Methods:**

We assessed cardiac function and cardiac mitochondria structure of SSM and IFM after 12 weeks of testosterone deficiency in male Wistar rats.

**Results and Discussion:**

Results show that low testosterone reduced myocardial contractility. Orchidectomy increased total left ventricular mitochondrial protein in the SSM, but not in IFM. The membrane potential, size and internal complexity in the IFM after orchidectomy were higher compared to the SHAM group. However, the rate of oxidative phosphorylation with all substrates in the IFM after orchidectomy was lower compared to the SHAM group. Testosterone replacement restored these changes. In the testosterone-deficient SSM group, oxidative phosphorylation was decreased with palmitoyl-L-carnitine as substrate; however, the mitochondrial calcium retention capacity in IFM was increased. There was no difference in swelling of the mitochondria in either group. These changes in IFM were followed by a reduction in phosphorylated form of AMP-activated protein kinase (p‐AMPK‐α), peroxisome proliferator‐activated receptor gamma coactivator 1‐alpha (PGC‐1α) translocation to mitochondria and decreased mitochondrial transcription factor A (TFAM). Testosterone deficiency increased NADPH oxidase (NOX), angiotensin converting enzyme (ACE) protein expression and reduced mitochondrial antioxidant proteins such as manganese superoxide dismutase (Mn-SOD) and catalase in the IFM. Treatment with apocynin (1.5 mM in drinking water) normalized myocardial contractility and interfibrillar mitochondrial function in the testosterone depleted animals. In conclusion, our findings demonstrate that testosterone deficiency leads to reduced myocardial contractility and impaired cardiac interfibrillar mitochondrial function. Our data suggest the involvement of reactive oxygen species, with a possibility of NOX as an enzymatic source.

## Introduction

Androgen deficiency commonly occurs in middle-aged and older men (1, 2) and is sometimes observed in younger men with hypothalamic-pituitary or testicular disorders (3, 4). Since the discovery of the androgen receptor in the heart and cardiomyocytes (5), evidence suggests that testosterone regulates heart function by direct actions on the myocardium. Supraphysiological testosterone levels have been associated with sudden cardiac death (6), cardiac hypertrophy (7), thrombosis (8), and may increase the rate of atherosclerosis progression leading to coronary artery disease and myocardial ischemia (9, 8). However, the mechanisms mediating testosterone’s myocardial actions remain unclear.

Clinical studies have shown that low levels of endogenous testosterone are also associated with cardiovascular diseases (10, 11) and a randomized trial showed that testosterone replacement improved functional exercise capacity, muscle strength, insulin and baroreflex sensitivity (2).

Testosterone plays an important role in the regulation of carbohydrate, fat and protein metabolism (12), which are strictly regulated by substrate availability and endocrine signaling (13). Therefore, changes in substrate availability, or testosterone levels could affect the supply of ATP from mitochondria. Since the heart has a limited storage capacity for ATP and very high-energy demand, the heart must continually generate ATP at a high rate to sustain contractile function, basal metabolic processes, and ionic homeostasis (14). Due to the intimate connection between oxidative metabolism and myocardial contractility, changes in mitochondrial function could result in reduced oxidative phosphorylation and contractility (15, 16).

In terms of energy production, cardiac cells contain two spatially distinct mitochondrial subpopulations: the subsarcolemmal subpopulation (SSM), located just below the sarcolemma and the interfibrillar subpopulation (IFM) which is located between the myofibrils (17–19). These two mitochondrial subpopulations not only differ in terms of their location, but their biochemistry (20). The literature suggests that SSM produces ATP to maintain the active transport of electrolytes and metabolites across the cell membrane, while IFM produce ATP to maintain the contractile function due to its location near the contractile machinery (17). Several studies demonstrated that the two subpopulations respond differently to physiological or pathological stimuli (17). Our hypothesis is that testosterone deficiency reduces cardiac and mitochondrial function in both subpopulations that are dependent on oxidative stress.

The present study aims to assess (1) cardiac contractility, (2) mitochondrial function, (3) oxidative phosphorylation, (4) morphology and mitochondrial membrane potential, and (5) permeability transition pore opening in the two subpopulations of cardiac mitochondria, thus providing new information on the effect of testosterone deficiency on cardiac contractility and mitochondrial function.

## Materials and methods

Male Wistar rats (250 g,12 weeks old) were acquired from the Federal University of Espirito Santo (UFES), Brazil and randomly divided into 3 groups (n = 30) as follows: a) SHAM (control) - The animals of this group underwent the surgical procedure (anesthesia and surgical incision) without orchidectomy; b) OQT Group (orchidectomy) – This group underwent surgery with radical orchidectomy (bilateral) and c) OQT + T Group (orchidectomy + testosterone). These orchidectomized animals were treated with testosterone (2 mg/kg i.m. daily) or placebo as previously described (21). In order to understand the influence of oxidative stress, another group of OQT rats (n = 8) was treated with apocynin (1.5 mM in drinking water) for 12 weeks.

After 12 weeks of treatment, all rats were anesthetized with urethane (1.2 g.kg^-1^). The thorax was opened, and blood was collected from the left ventricle (LV) and immediately placed on ice and centrifuged to obtain serum. The heart was removed, and the left and right ventricle removed and weighed. Sections of each ventricle were taken for biochemical analysis and stored at −80°C. The remaining cardiac tissue was used for mitochondrial isolation as described below. The liver, lungs, seminal vesicles, epididymal and retroperitoneal fat were also removed and weighed. The care and use of the laboratory animals was performed in accordance with *NIH* guidelines and the animal ethics committee of the Federal University of Espirito Santo (CEUA-UFES number: 072/2012) approved all procedures.

### Preparation of left ventricle papillary muscles

The heart was quickly excised, and the posterior papillary muscle was secured utilizing a set of rings. One of these ends was affixed to maintain stability, while the other was linked to a force transducer (TSD125 – Byopac Systems, Inc; CA), which was further linked to an amplifier (DA100C Byopac Systems, Inc; CA). Both the transducer and the amplifier were housed within a glass container brimming with 20 mL of nutrition solution with specific proportions of electrolytes and glucose (NaCl 120 mM; KCl 5.4 mM; CaCl2 1.25 mM; MgCl2 1.2 mM; NaH2PO4 2 mM; Na2SO4 1.2 mM; NaHCO3 18 mM and glucose 11 mM). This solution was subjected to a 95% O2 and 5% CO2 mixture at 30°C, pH 7.4. Subsequently, the papillary muscles underwent electrical stimulation provided by a pair of silver electrodes, which delivered rectangular pulses of 15 ms duration at a voltage 1.5 times the threshold (10 – 20 mV, 0.5 Hz). The papillary muscles were stretched to the point of maximum active tension muscle length (Lmax). Following this, a stabilization period of 30 minutes was allowed before the beginning of the experimental protocols. Data was captured using an acquisition system (MP100 Byopac Systems, Inc; CA), registering at an impressive rate of 500 samples per second. At the end, the papillary muscles were weighed, and the developed isometric contraction force was adjusted according to the weight of the muscles. These included the isometric contraction, presented as force expressed in grams per milligram of muscle weight (F; g/mg), the activation and relaxation times (time from contraction initiation to maximum strength peak and maximum peak time to 50% of isometric relaxation respectively), as well as values obtained from positive and negative force time derivatives (+ and - dF/dt; g/mg/s). The assessment of inotropic response to calcium was analyzed using a calcium concentration-response curve, applying varied concentrations of extracellular calcium: 0.62, 1.25, 2.5, and 3.75 mM CaCl_2_ in the perfusion solution. The resulting force variation was weight-corrected for the muscles and expressed in g/mg.

### Mitochondrial isolation

SSM and IFM were isolated according to the method of Palmer et al. (20), with minor modification (19 – 22). Briefly, the LV was rinsed in ice-cold Chappel-Perry buffer (100 mM KCl, 50 mM MOPS, 5 mM MgSO_4_, 1 mM ATP, 1 mM EGTA, 2 mg/ml BSA), blotted dry and then weighed. The ventricles were minced and homogenized in 1:10 (wt/vol) ice-cold Chappel-Perry buffer. The homogenates were centrifuged at 700 × g for 10 min. The supernatant containing SSM was extracted and centrifuged again at 10,000 x g to isolate SSM. The remaining pellet from the 700 x g spin was resuspended in KCl-MOPS-EGTA buffer containing 100 mM KCl, 50 mM MOPS, and 0.5 mM EGTA at pH 7.4, and treated with trypsin (5 mg/g) for 10 min at 4°C. The samples were incubated with trypsin inhibitor (albumin) and spun down at 700 x g for 10 min. The IFM-containing supernatant was then spun down at 10,000 x g for 10 min. The pellets were washed twice in ice-cold Chappel-Perry buffer and spun down at 10,000 x g for 10 min and then resuspended in ice KME buffer. The concentration of mitochondrial protein was measured by the Lowry method using bovine serum albumin as a standard.

### Mitochondrial respiration

Mitochondrial respiration was assessed in both IFM and SSM as described previously (18 – 23). Isolated mitochondria (0.5 mg/mL) were respired in respiration buffer containing 100 mM KCl, 50 mM 3-(N-morpholino)propanesulfonic acid buffer (MOPS), 5 mM KH_2_PO_4_, 1 mM EGTA and 1mg/mL BSA/Fraction V, using a polarographic oxygen-sensing systems for measurement of oxygen consumption in liquid suspension (Qubit System, Kingston, ON, Canada). States 3 and 4 respiration were measured with glutamate + malate (10 and 5 mM, respectively), pyruvate + malate (10 and 5 mM, respectively), palmitoyl-L-carnitine (40 µM), succinate (20 mM) + rotenone (7.5 µM) or ascorbate (0.2 mM) + N,N,N’,N’-tetramethyl-p-phenylenediamine (TMPD 0.5 mM) to assess respiration through the electron transport chain (ETC), exclusively. State 3 respiration (ADP-stimulated) was measured in the presence of 2 mM ADP. State 4 respiration (ADP-limited) was assessed after ADP consumption. Respiratory control ratio, the ratio of state 3 to state 4 was calculated to assess the control of oxygen consumption by phosphorylation. The ratio of ADP added in the chamber to the total amount of oxygen consumed in state 3 (ADP:O ratio) was calculated as an index of the efficiency of oxidative phosphorylation.

### Mitochondrial size and membrane potential

Mitochondrial size and membrane potential were measured as previously described (24, 19). Briefly, isolated IFM and SSM were stained with MitoTracker Green FM (Molecular Probes, Carlsbad, CA) and assessed using a flow cytometer (FACSCanto II, BD Biosciences). To confirm differences in absolute mitochondria size, we included size calibration beads composed of microsphere suspensions ranging in size from 0.5 to 6 µm to serve as reliable size references. The arithmetic mean output from the forward scatter detector was used as an index of mitochondrial size. For membrane potential, mitochondria were incubated with 5,5’,6,6’-tetrachloro-1,1’,3,3’-tetraethylbenzimidazol carbocyanine iodide (JC-1) (Molecular Probes, Carlsbad, CA) at a final concentration of 0.3 µM. The shift to orange is due to dye aggregates forming upon polarization, causing a shift in emitted light from 530 nm (green) to 590 nm (orange).

### Mitochondrial permeability transition pore opening

Ca^2+^-induced MPTP was evaluated in SSM and IFM from LV myocardium using previously described method (18, 19). The capacity for Ca^2+^ uptake was evaluated in isolated mitochondria by following the extramitochondrial [Ca^2+^] during a progressive, escalating exposure to Ca^2+^. Briefly, using a 96-well fluorometric plate reader at 37°C, 250 µg/mL of mitochondrial protein was suspended in calcium free buffer (100 mM KCl, 50 mM MOPS, 5 mM KH_2_PO_4_, 1 mM MgCl_2_, 5 mM EGTA) with glutamate + malate as substrate (5 and 2.5 mM). A bolus injection of 25 nmol free Ca^2+^ was injected every 7 min, and extramitochondrial [Ca^2+^] was recorded every 40-s using 750 nM Calcium Green-5N.

### Hormonal assay

Serum levels of testosterone and estrogen were measured according to the manufacturer. Testosterone levels were measured using the Testosterone ELISA kit (#29K116 42K034DRG Instruments GmbH, Germany). The assay detection limit for testosterone was 0.083 ng/mL. The intra-assay coefficient of variation for each assay was between 3.28% and 4.16%. The inter-assay coefficient of variation for each assay was between 4.73% and 9.94%. Estrogen levels were measured using the Estrogen ELISA kit (#42K026-3DRG Instruments GmbH,Germany) (25, 26). The assay detection limit for estradiol was 10.60 pg/mL. The intra-assay coefficient of variation for each assay was between 8.70% and 9.23%. The inter-assay coefficient of variation for each assay was between 6.87% and 14.91%.

### Western blotting

Western blot was performed as previously described (27). Samples (25-80 µg for whole heart sample and 10-30 µg for isolated mitochondria) were separated using 7.5%, 10% or 12% SDS-PAGE. Proteins were transferred to nitrocellulose membranes and incubated with specific antibodies: goat polyclonal anti-Serca-2 (1:1000; Santa Cruz Biotechnology-USA), rabbit polyclonal anti-p-PLB^Ser16^ (1:2500; Bradrilla-UK), mouse monoclonal anti-PLB (1:3000; Santa Cruz Biotechnology-USA), rabbit polyclonal anti-PGC-1α (1:1000; Santa Cruz Biotechnology-USA), rabbit polyclonal anti-NRF1 (1:500; Santa Cruz Biotechnology-USA), mouse monoclonal anti-Mfn-1 (1:200; Santa Cruz Biotechnology-USA), mouse monoclonal anti-Mfn-2 (1:200; Santa Cruz Biotechnology-USA), rabbit polyclonal anti-p-AMPK-α (1:1000, Cell Signaling, USA), rabbit polyclonal anti-AMPK-α (1:1000, Cell Signaling, USA), mouse monoclonal anti-total OXPHOS (CI subunit NDUFB8, CII-30kDa, CIII-Core protein 2, CIV subunit I and CV alpha subunit, 1:2500, Abcam, USA), rabbit polyclonal anti-MCU (1:1000, from Sigma-Aldrich-USA), rabbit polyclonal anti-SOD-1 (1:6000; Sigma-Aldrich-USA), mouse monoclonal anti gp91phox (1:2500 from Transduction Laboratories-USA), rabbit polyclonal anti SOD-1 (1:2000, Sigma-Aldrich-USA), mouse monoclonal anti-Cyclophilin D (1:2000, Mitoscience, USA), mouse monoclonal anti-TFAM (1:1000, Santa Cruz Biotechnology-USA), mouse monoclonal anti-catalase (1:12000, Sigma-Aldrich-USA), rabbit polyclonal anti-SOD-Mn (1:5000, Millipore-USA), rabbit polyclonal anti-NOX-1 (1:1000, Sigma-Aldrich-USA), rabbit polyclonal anti-ACE-1 (1:200, Santa Cruz Biotechnology-USA).

After washing, the membranes were incubated with anti-mouse (1:5.000, Sigma-Aldrich-USA), anti-goat (1:5.000, Sigma-Aldrich-USA) or anti-rabbit (1:5000, Sigma-Aldrich-USA) immunoglobulin antibodies conjugated to horseradish peroxidase. The immunocomplexes were detected using ECL Prime (Amersham International-UK) and the bands were detected using a Chemidoc system (Bio-Rad, USA). The same membranes were then used to determine GAPDH expression using a mouse monoclonal antibody (1:5000; Santa Cruz Biotechnology-USA) or ponceau staining for isolated mitochondria.

### Hydrogen peroxide production

Hydrogen peroxide production in isolated mitochondrial subpopulations IFM and SSM was determined using the oxidation of the fluorogenic indicator Amplex Red (Invitrogen) in the presence of horseradish peroxidase as previously described (28). The concentrations of horseradish peroxidase and Amplex Red in the incubation were 0.1 unit/mL and 50μM, respectively, and detection of fluorescence was assessed on a Molecular Devices Flex Station 3 fluorescence plate reader (Molecular Devices, Sunnyvale, CA) with 530-nm excitation and 590-nm emission wavelengths. Standard curves were obtained by adding known amounts of H_2_O_2_ to the assay medium in the presence of the substrates amplex red and horseradish peroxidase. H_2_O_2_ production was initiated in mitochondria using glutamate + malate (G + M) and succinate + rotenone (S + R) as substrates.

### Protein oxidation detection

To identify carbonyl groups that are introduced into the amino acid side chains after oxidative modification of proteins, 2D-oxyblot analysis was performed (29). The derivative that was produced by reaction with 2,4-dinitrophenylhydrazine (DNPH) was immunodetected by an antibody specific to the attached DNP moiety of proteins using a commercial kit (Oxyblot, Millipore).

The DNPH derivatization was carried out on 20 μg of protein for 15 minutes according to the manufacturer’s instructions. One-dimensional electrophoresis was carried out on a 12% SDS/polyacrylamide gel after DNPH derivatization. Proteins were transferred to nitrocellulose membranes which were then incubated in the primary antibody solution (anti-DNP 1: 150) for 2 h, followed by incubation in the secondary antibody solution (1:300) for 1 h at room temperature. The washing procedure was repeated eight times within 40 minutes. Immunoreactive bands were visualized by enhanced chemiluminescence (ECL; Amersham Biosciences) and immunodetection was carried out using a ChemiDoc system (Bio-Rad). To determine specificity, the oxidized proteins provided by the kit were included as a positive control. Treatment of samples with a control solution served as a negative control to the DNPH treatment. As an additional control, the anti-DNP antibody was omitted.

### Statistical analysis

Values are shown as mean ± standard error of the mean (SEM). Differences were analyzed using one or two-way ANOVA followed by a Fisher test. P < 0.05 was considered significant.

## Results

### Ponderal measurements

As shown in **Table 1**, castration of male animals for 12 weeks did not change body weight, left or right ventricle weight, demonstrating that endogenous testosterone does not affect the heart mass. As expected, castration decreased seminal vesicle weight after 12 weeks when compared to SHAM and testosterone replacement was able to restore this value.

**Table 1:** Ponderal data from SHAM, OQT and OQT plus testosterone 12 weeks after castration and testosterone replacement therapy.

	groups		
	SHAM n=10	OQT n=10	OQT+T n=10
**Body weight (g)**	447 ± 48	429 ± 47	437 ± 31
**Left ventricle (g)**	0.8 ± 0.1	0.8 ± 0.1	0.8 ± 0.1
**Right ventricle (g)**	0.2 ± 0.04	0.2 ± 0.05	0.2 ± 0.03
**Liver (g)**	15 ± 1.5	12.9 ± 1.6	12.9 ± 1.1
**Lung (g)**	2.2 ± 0.3	2.3 ± 0.4	2.3 ± 0.3
**Epididymal fat (g)**	7.3 ± 2.9	5.4 ± 1.7	5.5 ± 1
**Retroperitoneal fat (g)**	9.9 ± 3.9	12.7 ± 4.1	10.4 ± 3.4
**Seminal vesicle (g)**	1.1 ± 0.2	0.1 ± 0.02*	0.7 ± 0.2#
**Tibia (mm)**	40.3 ± 1.1	39.8 ± 1.3	39.9 ± 0.5

Data are expressed as mean ± S.E.M. *P<0.05 compared to SHAM. #P<0.05 compared to OQT group.

### Testosterone and estrogen serum levels

Testosterone levels decreased after 12 weeks in OQT animals and (SHAM: 2.09 ± 0,18 ng/mL; OQT: 0,53 ± 0.17 ng/mL^*^ and OQT + T: 2.16 ± 0.32 ng/mL^#^ n = 6 animals per group) and testosterone replacement restored to the SHAM level. Estradiol was also measured and was not different between groups (SHAM: 71.6 ± 5.2 pg/mL; OQT: 76 ± 5 pg/mL; OQT + T: 68 ± 7.71 pg/mL n = 6 animals per group).

### Myocardial contractility

**Figure 1:**
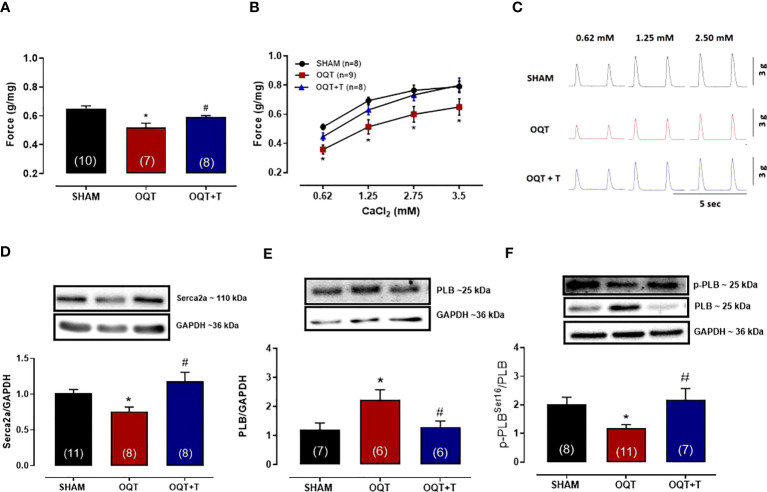
Testosterone deficiency decreases myocardial contractility after twelve weeks of hormone deprivation. The isometric force (g/g) in the left ventricular papillary muscles at baseline **(A)**, with different extracellular CaCl_2_ (0.62,1.25, 2.5 and 3.75 mM) concentrations **(B)**. Representative myograph tracing recorded from papillary muscles with different extracellular CaCl_2_ (0.62, 1.25 and 2.5 mM) concentrations from SHAM, OQT and OQT + T groups **(C)**. Western blot analyses of Serca2a protein expression **(D)**, total phospholamban **(E)** and phosphorylated phospholamban at Ser^16^ **(F)**. Results are presented as mean ± SEM values. Differences were analyzed using one-way ANOVA followed by Fischer *post-hoc* test. *P<0.05 compared to SHAM; #P<0.05 compared to OQT. Number of animals is indicated in parenthesis.

Myocardial contractility was analyzed using isolated papillary muscles at baseline and after exposure to different extracellular calcium concentrations. As expected, increasing extracellular calcium concentration resulted in a positive inotropic response in papillary muscles of all groups. However, the inotropic response to calcium at the concentrations of 0.62, 1.25, 2.5 and 3.75 mM were lower in the OQT group. (**Figures 1A–C**). Testosterone replacement prevented the reduction in contractile force because papillary muscles from OQT + T group presented similar inotropic responses as the SHAM group (**Figures 1A–C**). **Figure 1C** shows representative contractile responses recorded from papillary muscles paced at 0.5 Hz.

In myocardial cells, calcium handling depends upon Serca-2a and PLB function (30, 31). Serca2a and PLB protein expressions were measured in the hearts of our animals. As shown in **Figure 1D**, Serca2a protein expression was significantly decreased in the OQT group and this decrease was prevented by testosterone replacement. PLB protein expression was significantly increased in the OQT group and testosterone replacement prevented this increase in PLB protein expression (**Figure 1E**). We also measured phosphorylated phospholamban (P-PLB) at ser^16^, the main target for PKA phosphorylation. P-PLB levels were significantly reduced in the OQT group, indicating that PLB activity was enhanced in the OQT group (**Figure 1F**).

### Mitochondrial yield, size, and internal complexity

**Figure 2:**
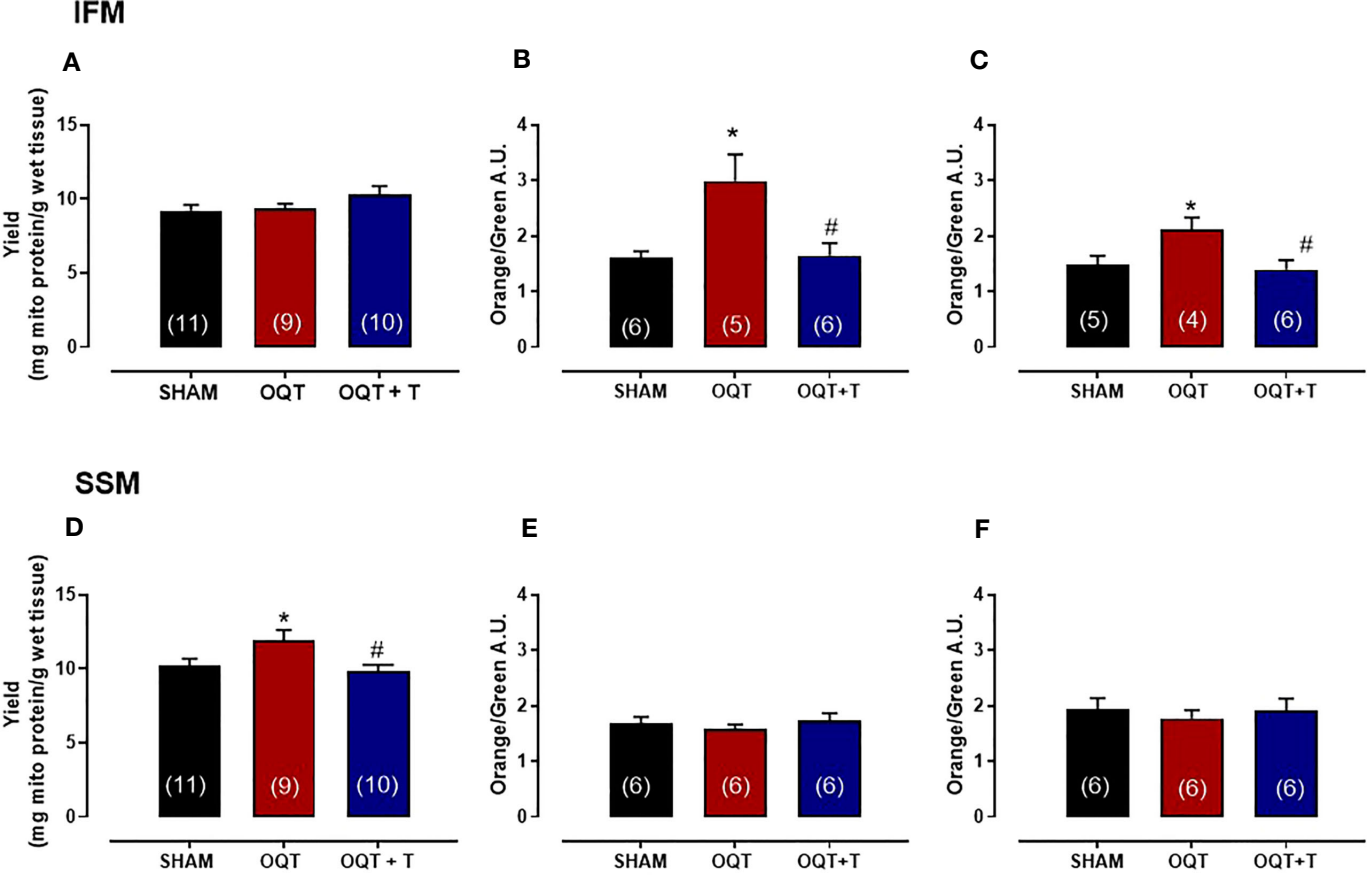
Testosterone deficiency increases mitochondrial membrane potential in interfibrillar mitochondria (IFM). Mitochondrial yield for isolated interfibrillar mitochondria (IFM) **(A)**, membrane potential with glutamate + malate (5 and 2.5 mM, respectively) **(B)** and membrane potential in the absence of substrate **(C)**. Mitochondrial yield for subsarcolemmal mitochondria (SSM) **(D)**, membrane potential with glutamate + malate (5 and 2.5 mM, respectively) **(E)** and membrane potential in the absence of substrate **(F)**. Isolated mitochondria stained with 5,5’,6,6’-tetrachloro-1,1’,3,3’-tetraethylbenzimidazol carbocyanine iodide (JC-1), which incorporates into intact mitochondria. Values for membrane potential are expressed as arbitrary units (AU). Serum testosterone levels (H) from SHAM, OQT and OQT + T groups. Results are presented as mean ± SEM values. Differences were analyzed using one-way ANOVA followed by Fischer *post-hoc* test. *P<0.05 compared to SHAM; #P<0.05 compared to OQT. Number of animals is indicated in parenthesis.

Yield refers to the amount of mitochondrial protein per gram of tissue. The yield for IFM was similar between the groups (**Figure 2A**); however, the yield for SSM was significantly increased in the OQT group when compared to SHAM (**Figure 2D**). Testosterone replacement prevented the increase in mitochondrial yield in the castrated animals. (**Figure 2D**).

To determine mitochondrial membrane potential and morphological differences between SHAM, OQT and OQT + T mitochondrial subpopulations, we used a gated flow cytometry method. Forward-scattered light (FSC) was used to estimate mitochondrial size, whereas assessment of side-scattered light (SSC) was used to estimate mitochondrial complexity, both of which were based on a logarithmic scale. Membrane potential of the mitochondrial subpopulations was analyzed by flow cytometry. Overall, baseline mitochondrial membrane potential (with endogenous substrate) was significantly increased in the IFM from OQT animals (**Figure 2B**), whereas the membrane potential was unchanged in the SSM (**Figure 2E**). We next incubated the samples with glutamate + malate (10 and 5 mM, respectively) to stimulate the ETC. While there was an initial increase in membrane potential in all groups when the substrates were added, membrane potential was significantly increased in the IFM, but not the SSM from the OQT groups (**Figures 2C, F**. For both substrates, the increase in IFM membrane potential was prevented by testosterone replacement (**Figures 2B, C**).

**Figure 3:**
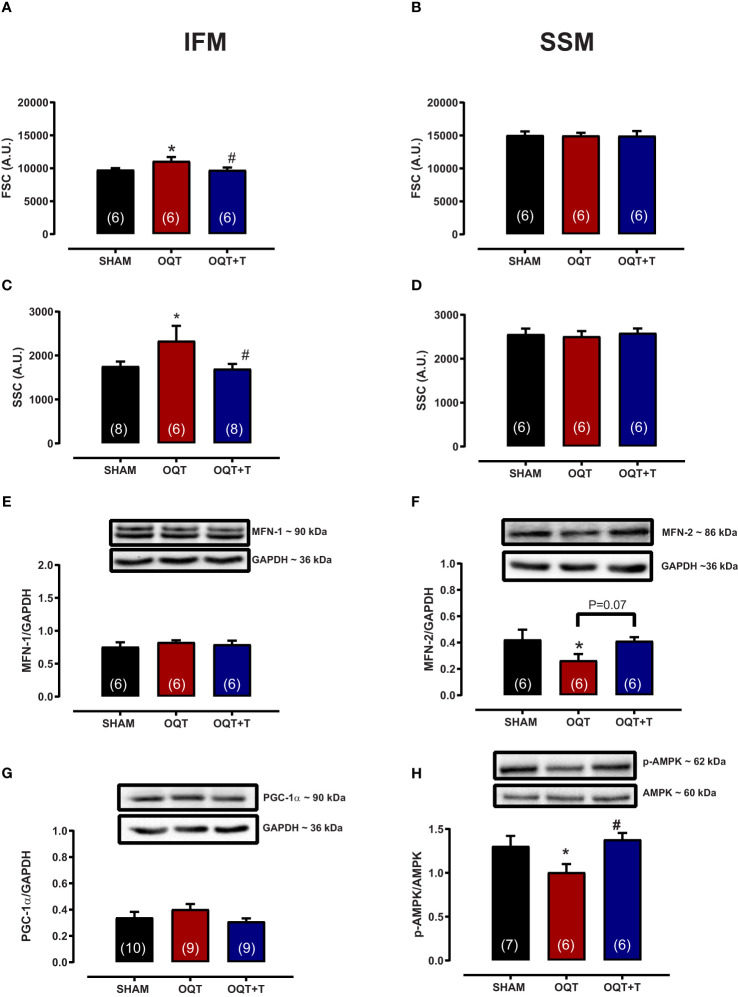
Testosterone deficiency increases interfibrillar mitochondrial internal complexity and size. Determination of the relative size **(A, B)** and internal complexity **(C, D)** of distinct mitochondrial subpopulations IFM and SSM using flow cytometric analyses. Western blot for mitofusion-1 **(E)**, mitofusion-2 **(F)**, PGC-1α **(G)**, and p-AMPK/AMPK **(H)** in whole heart tissue from SHAM, OQT and OQT+T groups. Results are presented as mean ± SEM values. Differences were analyzed using one-way ANOVA followed by Fischer *post-hoc* test. *P<0.05 compared to SHAM; #P<0.05 compared to OQT. Number of animals is indicated in parenthesis.

Mitochondrial morphology was assessed by flow cytometry to show relative morphological differences between the two subpopulations and confirm the success of the isolation. SSM were larger in size (FSC) and possessed greater internal complexity (SSC) compared with IFM, which were smaller and more compact (**Figures 3A–D**). Castration significantly increased the size and complexity of IFM compared to SHAM (**Figures 3A, C**). These increases were prevented by testosterone replacement. Conversely, the size and complexity of SSM were not affected by castration (**Figures 3B, D**).

To further investigate the mechanisms involved in mediating the changes in mitochondrial morphology, we developed western blots for mitofusins 1 and 2 which are involved in the regulation of mitochondrial fusion. **Figures 3E, F** show the expression of mitofusins 1 and 2, respectively. Mfn-1 protein expression was not altered between the groups. Mfn-2 was decreased significantly in the OQT group (**Figure 3F**). As mitochondrial biogenesis is regulated by PGC-1α and PGC-1α translocation to mitochondria is regulated by AMPK-α, we also measured the expression and phosphorylation of these two proteins. PGC-1α protein expression was not altered after twelve weeks of hormone deprivation (**Figure 3G**), whereas AMPK-α protein phosphorylation was decreased in the OQT group and restored to control levels by testosterone replacement (**Figure 3H**).

### Mitochondrial respiration

To assess the effects of testosterone deficiency on mitochondrial bioenergetics in the IFM and SSM, of the SHAM, OQT and OQT + T groups, we measured mitochondrial respiration with different substrates (glutamate + malate, pyruvate + malate, palmitoyl-L-carnitine, succinate + rotenone and ascorbate + TMPD) that use distinct oxidative pathways, mitochondrial transport and provide specific substrates to Complex I, Complex II and Complex IV.

**Figure 4:**
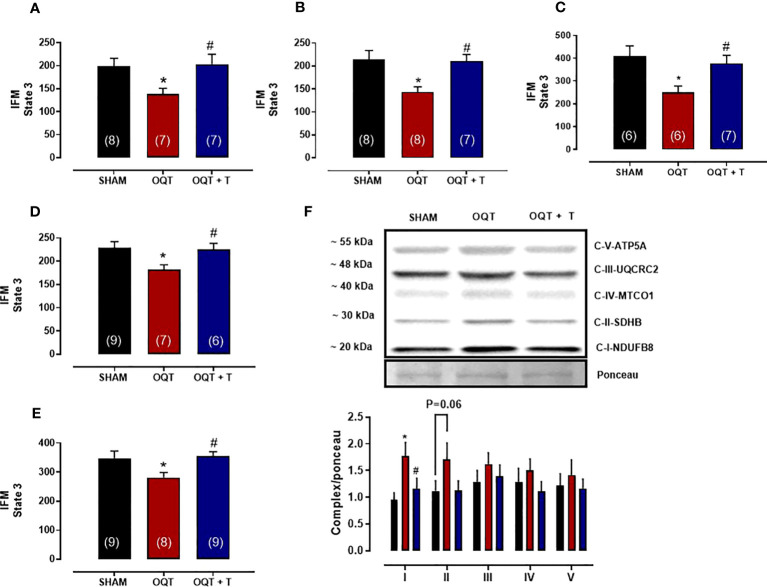
Testosterone deficiency decreases oxidative phosphorylation in interfibrillar mitochondria (IFM). Individual mitochondrial subpopulations were isolated from SHAM, OQT and OQT+T and polarographic measurements were performed to index oxygen consumption under state 3 and 4 respiration conditions. Respiration of individual electron transport chain (ETC) complexes was defined as the rate of oxygen consumed in the presence of specific substrates [glutamate and malate (complex I) **(A)**, β-oxidation **(B)**, ascorbate and N,N,N′,N′-tetramethyl-p-phenylenediamine (complex IV) **(C)**, pyruvate + malate **(D)** and succinate (complex II) **(E)**. All respiration rates are expressed in atoms O/mg mitochondrial protein^−1^· min^−1^. Western blot for total OXPHOS in IFM **(F)**. Results are presented as mean ± SEM values. Differences were analyzed using one-way ANOVA followed by Fischer *post-hoc* test. *P<0.05 compared to SHAM; #P<0.05 compared to OQT. Number of animals is indicated in parenthesis.

The maximal rate of mitochondrial respiration (State 3) in IFM expressed as nanomoles of oxygen per mg of mitochondrial protein was lower in the OQT group when compared to SHAM and OQT+T with glutamate + malate, pyruvate + malate, rotenone + succinate, palmitoyl-L-carnitine and ascorbate + TMPD as substrates (**Figures 4A–E**). To further investigate if the reduction in state 3 in the OQT group was due to low expression of the ETC complexes, we developed western blots for complex I-V, which showed that testosterone deficiency increased complex I and II protein expression in IFM (**Figure 4F**). These increases were prevented by testosterone replacement.

**Figure 5:**
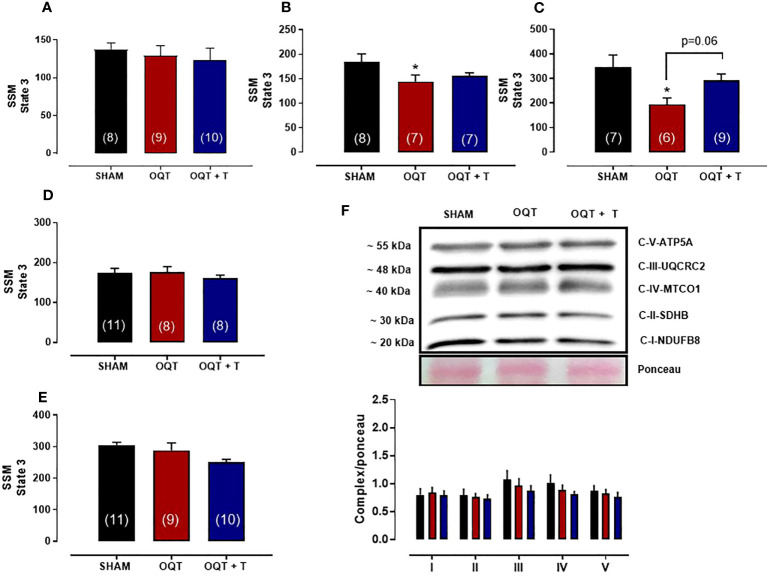
Testosterone deficiency decreases oxidative phosphorylation in subsarcolemmal mitochondria (SSM). Individual mitochondrial subpopulations were isolated from SHAM, OQT and OQT+T and polarographic measurements were performed to index oxygen consumption under state 3 and 4 respiration conditions. Respiration of individual electron transport chain (ETC) complexes was defined as the rate of oxygen consumed in the presence of specific substrates [glutamate and malate (complex I)] **(A)**, β-oxidation **(B)**, ascorbate and N,N,N′,N′-tetramethyl-p-phenylenediamine (complex IV) **(C)**, pyruvate + malate **(D)** and succinate (complex II) **(E)**. All respiration rates are expressed in atoms O/mg mitochondrial protein^−1^· min^−1^. Western blot for total OXPHOS in subsarcolemmal mitochondria **(F)**. Results are presented as mean ± SEM values. Differences were analyzed using one-way ANOVA followed by Fischer *post-hoc* test. *P<0.05 compared to SHAM. Number of animals is indicated in parenthesis.

We also measured state 3 with all substrates in SSM. State 3 was reduced with pyruvate + malate (**Figure 5B**) and rotenone + succinate (**Figure 5C**), but not with glutamate + malate (**Figure 5A**), palmitoyl-L-carnitine (**Figure 5D**) or ascorbate + TMPD (**Figure 5E**). Western blots for complex I-V did not reveal any differences in protein expression for the SSM (**Figure 5F**).

### Mitochondrial permeability transition pore opening

Two standardized methods were used to assess Ca^2+^-induced MPTP in LV mitochondria. MPTP was assessed from mitochondrial swelling induced by high [Ca^2+^] as reflected by the decrease in absorbance at 540 nm following the addition of a bolus of Ca^2+^ to isolated mitochondria. Measurement of baseline absorbance before the addition of Ca^2+^ was performed to determine basal values. There was a decrease in absorbance with addition of either 100 or 500 nmol Ca^2+^/mg protein in all groups, but no difference was found between the groups (data not shown).

**Figure 6:**
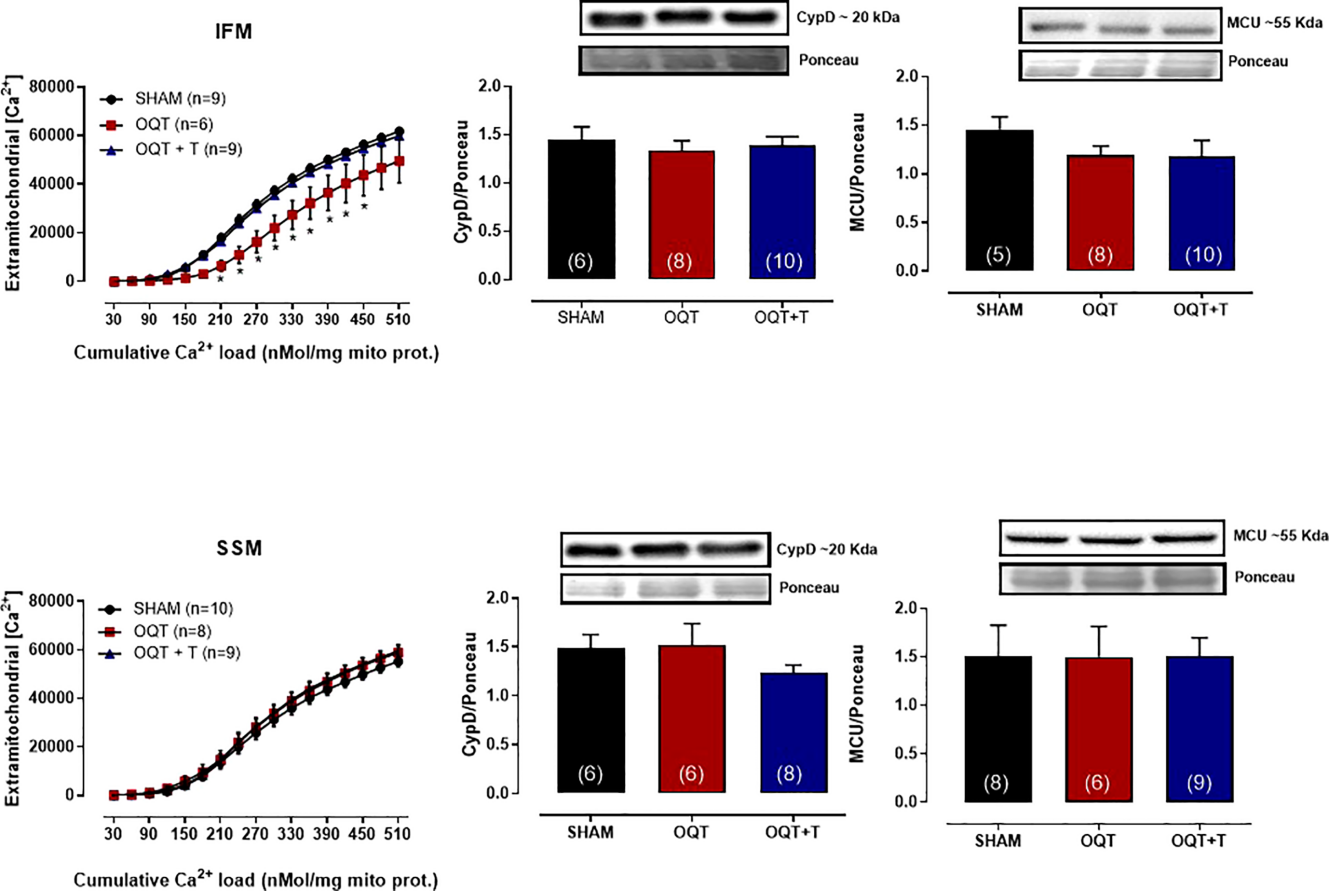
Testosterone deficiency increases mitochondrial calcium retention capacity in interfibrillar mitochondria. Calcium-retention capacity of the two distinct mitochondrial subpopulations isolated from SHAM, OQT and OQT + T. Mitochondria were primed for mitochondrial permeability transition pore (MPTP) opening with a progressive exposure to (Ca^2+^). 25 nmol free Ca^2+^ was injected every 7 min, and extramitochondrial [Ca^2+^] was recorded every 2 s using 750 nM Calcium Green-5N. Western blot analyses for cyclophilin D and mitochondrial uniporter (MCU) in interfibrillar mitochondria (top graphs) and subsarcolemmal mitochondria (bottom graphs). Results are presented as mean ± SEM values. Differences were analyzed using one and two-way ANOVA followed by Fischer *post-hoc* test. *P<0.05 compared to SHAM. Number of animals is indicated in parenthesis.

Ca^2+^-induced MPTP was also assessed by measuring the ability of isolated mitochondria to take up added Ca^2+^. As shown in **Figure 6** (top panel), IFM from the OQT group had significantly enhanced Ca^2+^ retention capacity compared to the SHAM and OQT + T groups. The Ca^2+^ uptake in SSM was the same in all groups (**Figure 6**, bottom panel).

To further elucidate whether these alterations in mitochondrial Ca^2+^ retention capacity were due to changes in cyclophilin D or MCU, we developed western blots for these two proteins. As shown in **Figure 6**, there were no differences in protein expression in either IFM (top panel) or SSM (bottom panel).

### Mitochondrial antioxidant enzymes, TFAM and PGC-1α translocation to mitochondria

**Figure 7:**
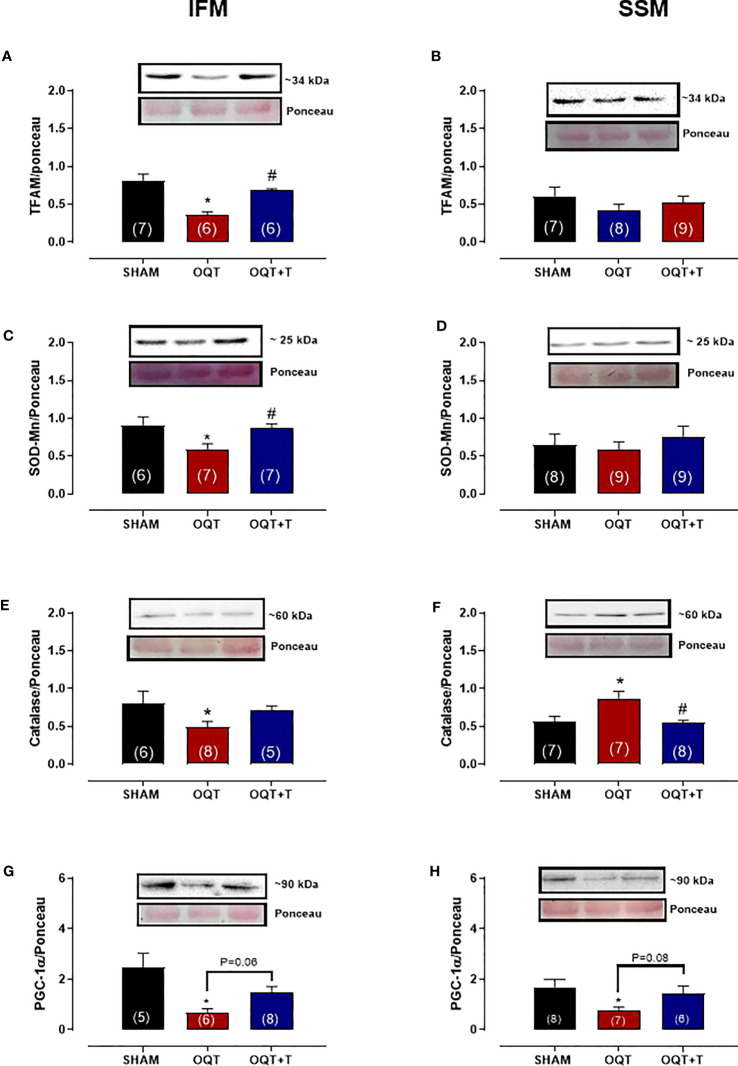
Testosterone deficiency decreases interfibrillar mitochondrial antioxidant enzymes and transcription factors. Western blot analyses for mitochondrial transcription factor A (TFAM) **(A, B)**, mitochondrial superoxide dismutase (SOD-Mn) **(C, D)**, mitochondrial catalase **(E, F)** and mitochondrial peroxisome proliferator-activated receptor γ coactivator 1α **(G, H)**. Results are presented as mean ± SEM values. Differences were analyzed using one-way ANOVA followed by Fischer *post-hoc* test. *P<0.05 compared to SHAM; #P<0.05 compared OQT. Number of animals is indicated in parenthesis.

PGC-1α trans-activates nuclear respiratory factor 1, which in turn, activates mtDNA transcription factor A (TFAM) that regulates mtDNA transcription and replication. Subsequently, NRF-1 promotes transcription of mitochondrial transcription factor TFAM, which targets mtDNA-encoded genes. TFAM protein expression was significantly reduced after castration in IFM (**Figure 7A**), but not in SSM (**Figure 7B**). Again, the reduction in TFAM could be prevented by testosterone replacement (**Figure 7A**).

In the IFM, the expression of mitochondrial Mn-SOD (**Figure 7C**) and catalase (**Figure 7E**) were significantly decreased after castration and this reduction was prevented by testosterone replacement. In SSM, mitochondrial SOD-Mn expression (**Figure 7D**) was unchanged by castration, while catalase levels (**Figure 7F**) were significantly increased. The PGC-1a protein expression decreased in both IFM (**Figure 7G**) and SSM (**Figure 7H**) OQT group.

### Orchidectomy increases NADPH oxidase protein expression and mitochondrial protein oxidation

**Figure 8:**
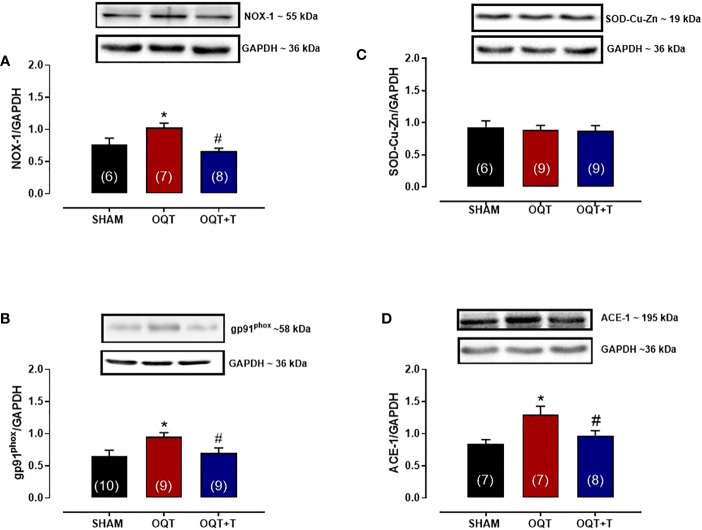
Testosterone deficiency increases NADPH oxidase (NOX-1), gp91phox and ACE-1 protein expressions. Western blot analyses for NOX-1 **(A)**, gp91^phox^ **(B)**, superoxide dismutase Cu -Zn **(C)**, angiotensin converting enzyme 1 **(D)** in cardiac tissue from SHAM, OQT and OQT + T groups. Results are presented as mean ± SEM values. Differences were analyzed using one-way ANOVA followed by Fischer *post-hoc* test. *P<0.05 compared to SHAM; #P<0.05 compared OQT. Number of animals is indicated in parenthesis.

**Figure 9:**
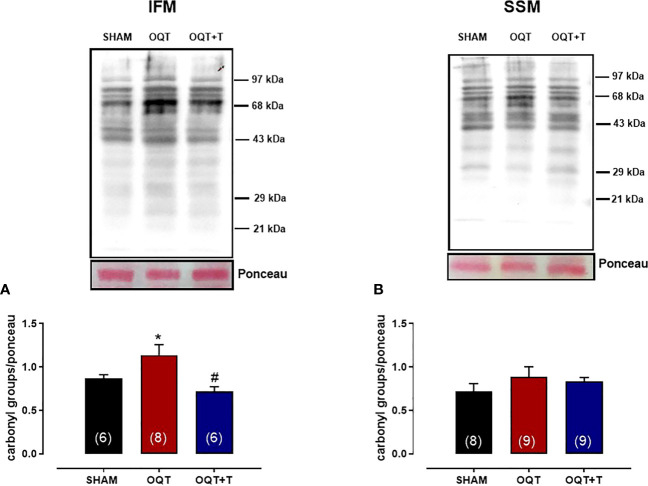
Testosterone deficiency increases interfibrillar mitochondrial protein oxidation. Immunodetection of carbonyl groups that are introduced into the amino acid side chain after oxidative modification of proteins in the interfibrillar mitochondria **(A)** and subsarcolemmal mitochondria **(B)**. Results are presented as mean ± SEM values. Differences were analyzed using one-way ANOVA followed by Fischer *post-hoc* test. *P<0.05 compared to SHAM; #P<0.05 compared OQT. Number of animals is indicated in parenthesis.

It is well known that NADPH oxidase transfers an electron to molecular oxygen to form superoxide anion. We examined the role of NADPH oxidase isoforms and mitochondrial protein oxidation in the hearts from our groups. Western blots showed significant increases in NOX-1 and gp91^phox^ in the hearts of the OQT group and testosterone replacement restored this increase to SHAM levels (**Figures 8A, B**). We next analyzed protein expression for SOD-Cu-Zn (**Figure 8C**) and angiotensin converting enzyme (ACE-1) (**Figure 8D**). SOD-Cu-Zn in the heart was not changed after castration; however, ACE-1 protein expression was significantly increased in the OQT group. This increase was blocked by testosterone replacement. As reactive oxygen species can promote protein carbonylation, we developed western blots to detect DNPH-derivatized proteins in both mitochondrial subpopulations. As shown in **Figures 9A, B**, orchidectomy significantly increased mitochondrial protein carbonylation in IFM, but not in SSM. The increased protein carbonylation in IFM was prevented by testosterone replacement (**Figure 9A**).

### Apocynin treatment prevents OQT induced myocardial contractility dysfunction

**Figure 10:**
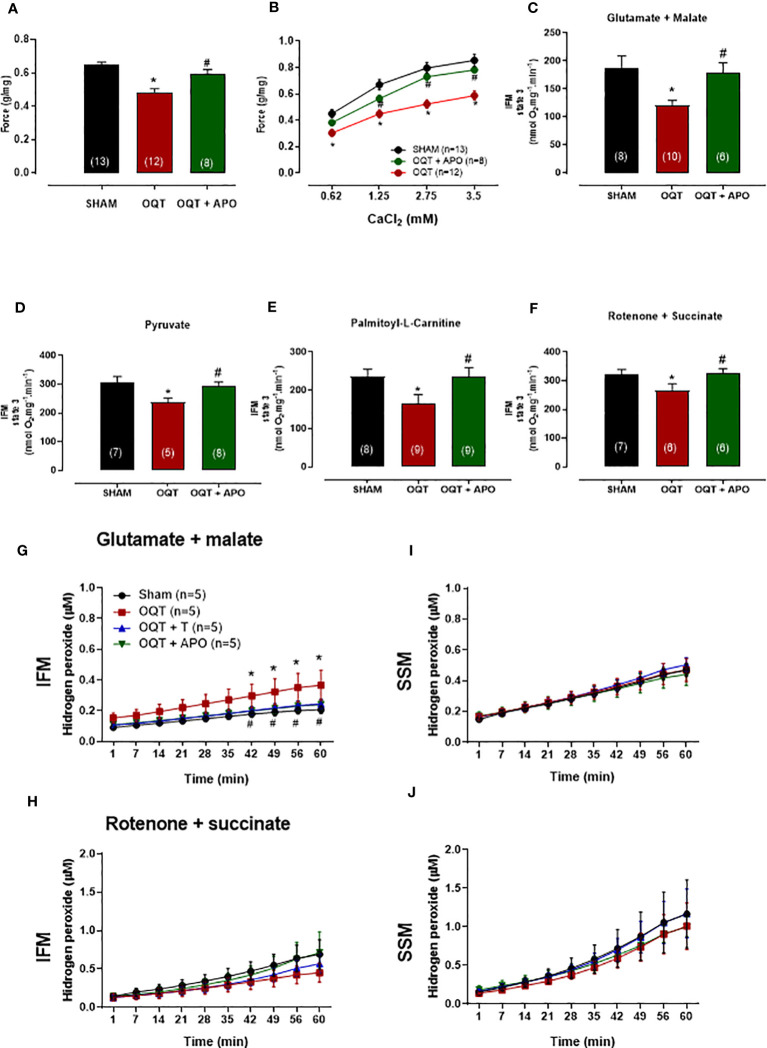
Oxidative stress on myocardial contractility was analyzed using apocynin in drinking water. Apocynin prevented the reduction of myocardial contractility **(A, B)** and mitochondrial function **(C–F)**. The isometric force (g/g) in the left ventricular papillary muscles at baseline **(A)**, and at different extracellular CaCl_2_ (0.62, 1.25, 2.5 and 3.75 mM) concentrations **(B)**. Individual mitochondrial subpopulations were isolated from SHAM, OQT and OQT+T and polarographic measurements were performed to index oxygen consumption under state 3 respiration conditions. Respiration of individual electron transport chain (ETC) complexes was defined as the rate of oxygen consumed in the presence of specific substrates [glutamate and malate (complex I) **(C)**, pyruvate **(D)**, β-oxidation in the presence of specific substrate palmitoyl-L-carnitine **(E)**, and rotenone + succinate (complex II) **(F)**. All respiration rates are expressed in Atoms O.mg mitochondrial protein^−1^· min^−1^. Testosterone deficiency increases hydrogen peroxide production in intermyofibrillar mitochondria. **(G, H)** heart mitochondria for isolated interfibrillar mitochondria (IFM) and **(I, J)** heart mitochondria for isolated subsarcolemmal mitochondria (SSM) subpopulations were added to the mixture of Amplex Red and basal H_2_O_2_ formation was measured (µM). Differences were analyzed using one-way ANOVA followed by Fischer *post-hoc* test. *P<0.05 compared to SHAM; #P<0.05 compared to OQT. Number of animals is indicated in parenthesis (n).

In order to understand the influence of oxidative stress, a separate group of OQT rats (n=8) was treated with apocynin (1.5 mM in drinking water) for 12 weeks after castration. Myocardial contractility was analyzed using isolated papillary muscles at baseline (**Figure 10A**) and with different extracellular calcium concentrations (**Figure 10B**). Increasing extracellular calcium concentration elicited a positive inotropic response in the papillary muscles of all groups; however, the inotropic response was significantly smaller in the OQT group. Treatment of the castrated rats with apocynin prevented the reductions in the inotropic responses to calcium (0.62, 1.25, 2.5 and 3.75 mM) (**Figures 10A, B**). Treatment with apocynin also prevented the castration-related decreases in state 3 oxidative phosphorylation, independent of substrate (**Figures 10C–F**).

### Apocynin treatment prevents OQT induced mitochondrial H_2_O_2_ production

The ability of mitochondria to generate H_2_O_2_ was evaluated in isolated interfibrillar mitochondria (IFM) subpopulations (**Figures 10G**, **H**) and subsarcolemmal mitochondria (SSM) subpopulations (**Figures 10I**, **J**) from the left ventricular (LV) myocardium using the amplex red assay. Testosterone deficiency resulted in an increase in hydrogen peroxide production in IFM (**Figure 10G**) when using the glutamate + malate substrate. Treatment with apocynin prevented oxidative stress-induced mitochondrial H_2_O_2_ production in the IFM subpopulation when using the glutamate + malate substrate.

## Discussion

This investigation assessed the effects of testosterone deficiency on myocardial contractility and the function of the spatially distinct subpopulations of cardiac mitochondria. Our data showed that orchidectomy significantly decreased myocardial contractility after twelve weeks of hormone deprivation. In addition, while the yield of SSM in the heart was increased after testosterone deprivation, the structure and function of these mitochondria was not substantially altered. In contrast, the reduction in testosterone elicited significant changes in the IFM, including increased membrane potential, mitochondrial size and internal complexity. Moreover, the rate of oxidative phosphorylation in the IFM was significantly reduced in the OQT animals independent of the substrate analyzed, for complex I, II and IV. IFM from OQT animals also had a higher Ca^2+^ retention capacity, implying that IFM from orchidectomized animals are less susceptible to permeability transition pore opening. Finally, we provided data using apocynin suggesting that oxidative stress is involved in the cardiac dysfunction and increased IFM protein oxidation produced by testosterone deficiency.

It is well known that testosterone plays a significant role in cardiac contraction coupling and that the lack of this hormone decreases myocardial contractility due to a decrease in Serca2a activity (32–35). Our data shows that orchidectomy reduces myocardial contractility in a time dependent manner. We previously showed that, myocardial contractility is preserved 8 weeks after orchidectomy in rats (36). However, in the present study, 12 weeks after orchidectomy, myocardial contractility was significantly reduced, corroborating previous findings in the literature (32). Furthermore, our data also show that phospholamban protein expression is increased and its phosphorylation at Ser^16^ (37) is reduced, indicating that Serca2a activity is reduced after 12 weeks of deficiency.

Myocardial force generation relies on the energy supplied by ATP, which is mainly produced by mitochondria. Approximately 95% of this ATP comes from oxidative phosphorylation processes and is delivered to consuming sites such as myosin-ATPase (60-70%), Serca2a (30-40%) and Na^+^/K^+^-ATPase (8, 27) [51). Efficient matching of energy production and consumption is crucial for beat-to-beat adaptation to changes in cardiac workload and involves important molecules and ions such as ADP and Ca^2+^ (38, 39). Furthermore, the fine-tuning of energy production and expenditure is also regulated by gene transcription (39). It has been hypothesized that IFM supply ATP for contraction, whereas SSM are involved primarily in providing ATP for active transport of electrolytes and metabolites across the sarcolemma (17) (20, 40, 41). Here we showed that testosterone deficiency induced IFM dysfunction after 12 weeks as evidenced by increased size, reduced bioenergetics and increased protein oxidation. The increase in mitochondrial size could be due to reduced Mfn-2 protein expression. Wang et al. also demonstrated that testosterone deficiency decreased Mfn-2 protein expression and corroborated our findings (21) showing that IFM have increase size and greater calcium retaining capacity. The literature demonstrates that knockout mice for Mfn-2 have larger mitochondria and greater calcium retention capacity (42).

Mitochondrial calcium signaling plays a key role in the regulation of cellular energy metabolism, cardiac excitation–contraction coupling and the generation of ATP for contraction. In the mitochondrial matrix, Ca^2+^ plays an important role in energetics by activating the F1/FO ATPase (43) and several Ca^2+^ sensitive dehydrogenases in the tricarboxylic acid cycle, including pyruvate dehydrogenase, 2-oxoglutarate (α-ketoglutarate) dehydrogenase, and the NAD^+^-linked isocitrate dehydrogenase (44, 45). Activation of these enzymes results in increased NADH production, which is critical for matching energy supply with demand during increased workload (46). The mechanism that triggers calcium influx across the inner mitochondrial membrane is the large negative membrane potential (-180 mV) that drives cytosolic calcium entry into the matrix (47). The higher membrane potential in the OQT group could be responsible for the increased calcium uptake we observed in the IFM. Furthermore, the higher calcium concentration in the matrix most likely does not increase NADH production due to the decreased oxidative phosphorylation and cytochrome c oxidase activity that has been demonstrated by us and others (48). It has been proposed that IFM generate ATP for the contractile machinery. Thus, it is possible that the reduced force generation we observed in the OQT animals is the result of the reduced state 3 activity found in this group. The lower activity of the electron transport chain could be due to posttranslational modification, such as oxidation which most likely, underlies the increase in carbonylation we observed in IFM.

PGC-1α is implicated in the regulation of the mitochondrial genome. Recently, it has been demonstrated that PGC-1α is located within mitochondria, where it co-localizes within the D-loop of mtDNA with mitochondrial transcription factor A (TFAM) (49). Furthermore, AMPKα phosphorylates PGC-1α, which in turn translocates to mitochondria and forms a complex with TFAM. TFAM also exists in subcellular compartments from which it can translocate to mitochondria. TFAM regulates nuclear DNA such as Serca2a expression in cardiomyocytes. The transcription of genes for mitochondrial ATP production and the genes for proteins that consume large amounts of ATP (Serca2a) are regulated by the same transcription factor. Oxidative stress decreases TFAM levels in both the nucleus and the mitochondria (39). Correspondingly, our results demonstrated that phosphorylated AMPK-α is reduced, while PGC-1α protein expression did not change in whole heart tissue after castration. Protein content was reduced in both mitochondrial subpopulations, indicating that PGC-1α translocation is reduced in our model. We also detected decreased TFAM protein expression in both IFM and SSM. Oxidative stress was also increased as indicated by NOX-1 and gp91^phox^ overexpression. It has been proposed that NADPH oxidase causes mitochondrial damage in aging and hypertrophic cardiomyocytes (50). NOX-2 also stimulates mitochondrial ROS by activating reverse electron transfer in mitochondria (51). Conversely, inhibition of mitochondrial ROS production improves cardiac and mitochondrial dysfunction in heart failure (28). Similarly, our data show that inhibition of NOX normalizes cardiac and mitochondrial function in the face of low testosterone.

The effects of testosterone deficiency on SSM function have been demonstrated previously (9); however, these effects are controversial (52), possibly due to the fact that previous studies only analyzed one mitochondrial subpopulation. The literature describes that cardiac cells contain discrete pools of mitochondria that are characterized spatially by their subcellular location (17). The effects of testosterone deficiency on IFM function have not been previously reported. One interesting aspect of our data is that catalase expression was increased in SSM but reduced in IFM. We also found that protein oxidation was increased in IFM but did not change in SSM. These findings could explain the slight dysfunction found in SSM. As previously reported, overexpression of mitochondrial catalase attenuates pressure overload-induced heart failure (53). One question that remains to be answered is why the two distinct subpopulations of mitochondria respond differently to the same stimulus. Further investigation is needed to answer this question.

The present research offers valuable insights but has some limitations. One such limitation is that using apocynin to analyze NOX lacks specificity for NOX enzymes. Apocynin has been widely recognized as a NOX inhibitor, but it can also interact with other enzymes or cellular components, potentially leading to off-target effects. Therefore, while apocynin can provide valuable insights into the overall involvement of NOX in a biological process, it cannot definitively establish the specific isoform or enzymatic source of ROS. The second important limitation is predominantly due to the inherent differences between human and rat physiology, rendering the translatability of the findings to humans uncertain. Differences span from metabolism, implying potential divergence in dose-response relationships, to ethical and logistical constraints limiting the scope of measures such as organ weight alterations. Confounding factors may also impact study outcomes. The age of animal subjects and strain-specific attributes may affect results, reflecting the dynamic nature of testosterone levels, as well as cardiac and mitochondrial functions. External factors such as housing conditions, diet, stress levels, and comorbid conditions, which are commonly present in humans suffering from low testosterone, like obesity and diabetes, might influence the results. These confounders may not be adequately accounted for in an animal study. While providing insightful preliminary data, these limitations necessitate cautious interpretation of the findings. For comprehensive understanding and clinical relevance, validation through carefully designed human clinical trials is indispensable.

In conclusion, our data show that low testosterone reduces cardiac contractile function. Moreover, our data suggests that the contractile deficit may reflect an oxidative stress-mediated decrease the ability of the IFM to supply ATP to the contractile filaments.

## Data availability statement

The raw data supporting the conclusions of this article will be made available by the authors, without undue reservation.

## Ethics statement

The animal study was reviewed and approved by the animal ethics committee of the Federal University of Espirito Santo (CEUA-UFES number: 072/2012).

## Author contributions

PL, KR, EAM, PR, RÁ, EM, JG, MS, and RR performed the experiments, analyzed the data, and discussed the results. IS and RR were involved in the study conception, data analyses and interpretation, discussing the results, and writing the paper. IS is the corresponding author. All authors contributed to the article and approved the submitted version.
